# Weber B Fracture of the Lateral Malleolus with Concomitant Anterior Talofibular Ligament Injury following an Ankle Supination Injury

**DOI:** 10.1155/2016/8035029

**Published:** 2016-05-29

**Authors:** Mohammed Khalid Faqi, Abdulla AlJawder, Fahad Alkhalifa, Ali H. Almajed

**Affiliations:** Department of Orthopedic Surgery, Bahrain Defence Force Hospital-Royal Medical Services, Riffa, Bahrain

## Abstract

The Lauge-Hansen (LH) classification attempts to predict patterns of ankle injuries based upon the preceding mechanism of injury. Although it is widely used in clinical practice, it has been criticized mainly due to numerous reports of cases conflicting the prediction system. Here, we report a case of a 32-year-old male who sustained a Weber B fracture of the lateral malleolus following a supination ankle injury, which was treated conservatively, following which the patient presented with ankle instability and was found to have concurrent anterior talofibular ligament tear. Critical review of the LH classification along with its shortcomings is discussed.

## 1. Introduction

The lateral ligaments of the ankle comprise the anterior talofibular ligament (ATFL), the calcaneofibular ligament (CFL), and the posterior talofibular ligament (PTFL). The ATFL attaches to the distal end of the fibula and the lateral surface of the talus bone, having its center approximately 10 mm above the apex of the lateral malleolus. It is the most commonly injured ligament in the ankle. ATFL injuries can present as an isolated tear or accompanied with avulsions of the lateral malleolus.

In a series of cadaveric studies in the 1950s, Lauge-Hansen replicated different patterns of injury mechanisms by fracturing amputated lower limb specimens, through which he described a classification system which attempts to predict patterns of ankle fractures and associated ligamentous injuries, based on the mechanism of injury [1–5].

According to Lauge-Hansen (LH) classification, type 1 supination-adduction injury can translate to either a lateral ligament injury (most commonly the ATFL) or a lateral malleolus transverse avulsion fracture. Type 2 supination-external rotation (SER) injury translates to an anterior inferior talofibular ligament (AITFL) injury followed by a short oblique lateral malleolus fracture.

In this case report, we present a patient with a severed ATFL (midsubstance tear) and a nondisplaced oblique fracture of the lateral malleolus at the left of the syndesmosis. The AITFL was intact. This fracture pattern does not translate to any class of the LH classification system. To the best of our knowledge, there are no reported cases in indexed literature of a lateral ankle instability following the removal of cast in a closed transsyndesmotic lateral malleolus fracture, with concomitant complete lateral ligament tear requiring intervention.

## 2. Case Report

A 32-year-old male presented to the accident and emergency department suffering from a twisting injury to his left ankle. His chief complaints were diffuse, left ankle pain and inability to weight bear. Further history revealed that a supination ankle injury was sustained during football practice. On examination, his left ankle was significantly swollen with maximal tenderness over the lateral malleolus. He had no tenderness on the medial malleolus and no wounds. Posterior tibial and dorsalis pedis pulses were felt and a neurological examination was normal.

**Figure 1:**
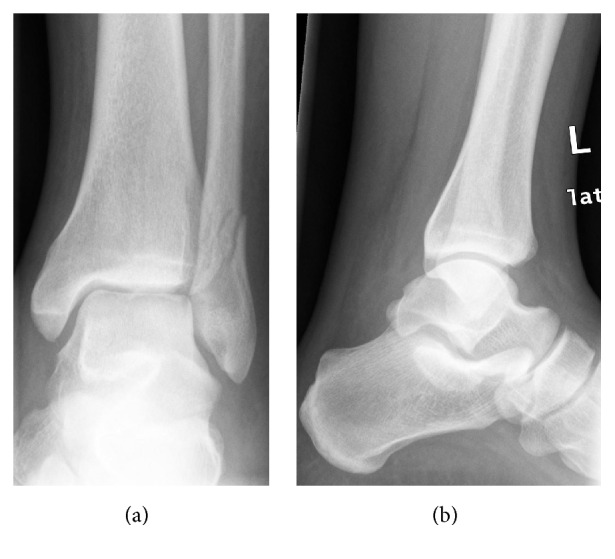
Plain (a) anteroposterior and (b) lateral radiographs of the left ankle on initial presentation revealing a nondisplaced, short, oblique fracture of the lateral malleolus, Danis-Weber B.

Plain anteroposterior and lateral radiographs of the left ankle revealed a nondisplaced, short, oblique fracture of the lateral malleolus, Danis-Weber B. No talar tilt, syndesmotic injury, or other fractures were noted (Figure 1). The patient was managed conservatively with a below knee full cast and was kept non-weight bearing. Repeated plain radiographs taken during a follow-up 2 weeks later showed no displacement. The patient was continued on the same treatment.

**Figure 2:**
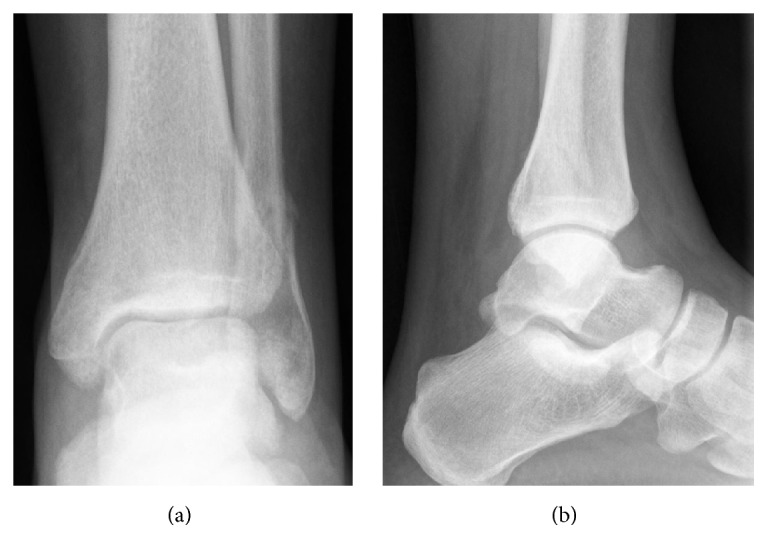
Plain (a) anteroposterior and (b) lateral radiographs of the left ankle at four weeks following injury, showing good alignment with callus formation at the fracture site.

Two weeks later, plain radiographs (AP and lateral) of the left ankle showed good alignment with callus formation at the fracture site (Figure 2). Four weeks later, the cast was removed and the patient started weight bearing, as tolerated. He then started physical therapy sessions for his left ankle for 3 months.

**Figure 3:**
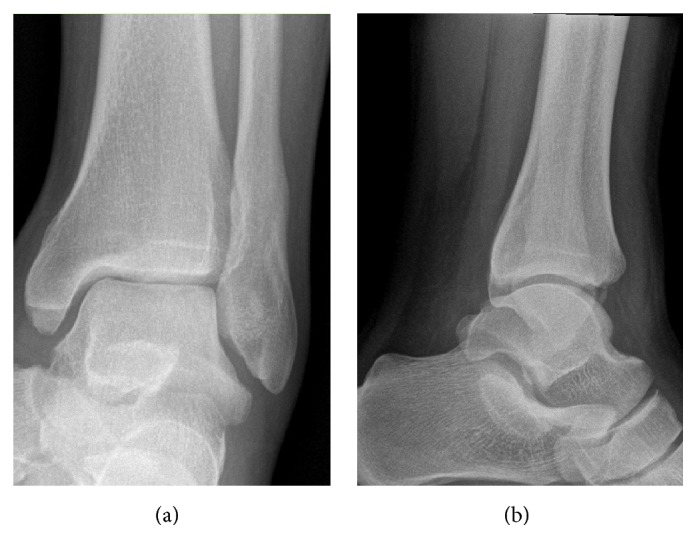
Plan (a) anteroposterior and (b) lateral radiographs of the left ankle showing good signs of healing of the lateral malleolus fracture following completing the physiotherapy rehabilitation.

**Figure 4:**
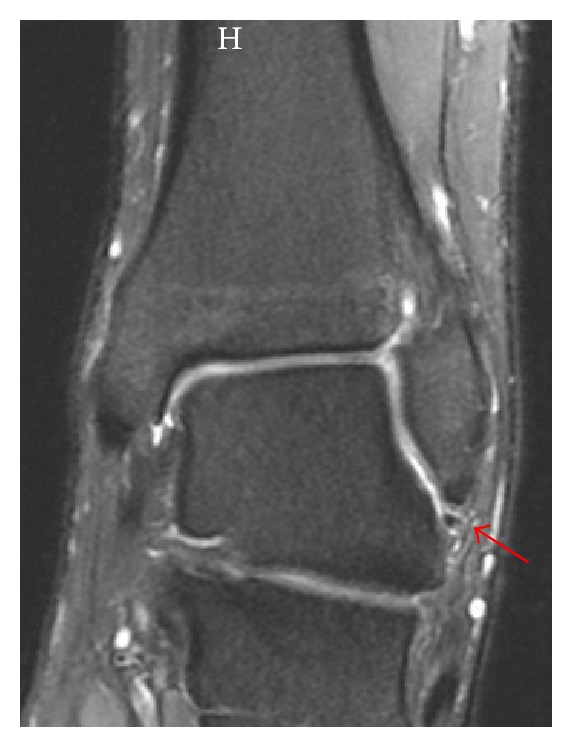
Coronal proton density fat saturated MR image showing an ATFL tear (arrow).

**Figure 5:**
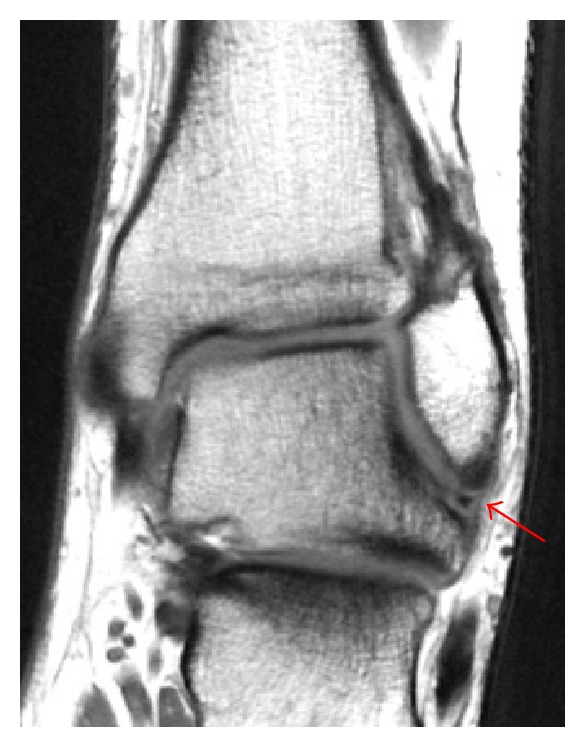
Coronal T1-weighted MR image showing an ATFL tear (arrow).

The patient was still complaining of pain over the anterolateral aspect of the left ankle with symptoms of ankle instability. On examination of the left ankle, anterior drawer test was positive and pain increased on dorsiflexion of the foot. Plain radiograph (Figure 3) showed a healing fracture, with no other findings. Magnetic resonance imaging (MRI) of the left ankle showed an ATFL midsubstance and complete tear (grade 3) and was otherwise normal (Figures 4 and 5).

With the impression of an anterolateral impingement syndrome of the left ankle and an ATFL tear, arthroscopic debridement and a modified Broström procedure were done under general anesthesia. The patient recovered with no complications. Postoperatively, the patient was kept on a full cast for 2 weeks and advised non-weight bearing. During the 3rd and 4th weeks, he was kept on a controlled ankle motion (CAM) boot from 10° dorsiflexion to 20° plantarflexion and started partial weight bearing. The CAM boot was increased to 20° and dorsiflexion to 40° plantarflexion during the 5th and 6th postoperative weeks. Finally, the patient was weaned off the CAM boot, progressing to full weight bearing at the end of the 6th week. With ankle physical therapy, his symptoms and ankle function gradually improved.

## 3. Discussion

In the past decade, the LH classification has been criticized in many studies. This is mainly because numerous cases were reported where the injury findings conflicted with the LH classification pattern of injury. Ever since, the reliability and reproducibility of the LH classification have been in question. However, regardless of its limitations, its importance lies in attempting to predict ankle ligament injuries not visualized on radiographs [6].

Gardner et al. investigated the accuracy of the LH classification in predicting the mechanism and ligament injury patterns of ankle fractures using magnetic resonance imaging (MRI) [7]. The authors found that 53% of the fractures had patterns of ligamentous injuries that did not correspond with the LH classification. Moreover, 24 patients (41%) were reported to have both ATFL injury and fracture of the fibula. However, the absence of clinical sequelae of ATFL injuries was considered due to the planned postoperative immobilization, although no documentation of outcomes after fracture treatment was available. No other reports of complete ligament rupture with concomitant fracture of the lateral malleolus were found in indexed literature. Conversely, in a larger cohort study of 300 ankle fractures with a similar design, Warner et al. reported that 94% of MRI readings were consistent with the LH classification. The authors conclude that the MRI is not as reliable as the LH classification in identifying ligament injuries. They attributed the pattern of lateral malleolus fracture with complete tear of the associated ligament as an overestimation in MRI images, that is, false positives. Of note are the injuries of all patients being classifiable by LH classification [8]. Similarly, Kumar et al. reported a superiority of radiographs compared to MRI in diagnosing ligament injuries in ankle fractures [9]. Hermans et al. analyzed 51 ankle fractures focusing on syndesmotic injuries and compared Weber, AO, and LH classifications with MRI findings. LH classification had a 92% correlation with the MRI. Of the 51 patients, 4 patients were not classifiable as they presented with a solitary medial malleolus fracture with an unknown collateral ligament status, that is, being unable to differentiate LH 1 from LH 2 (pronation and external rotation from pronation and abduction) [10].

Several cadaveric studies failed to fully validate the LH classification of injuries. Haraguchi and Armiger attempted to replicate LH pronation-external rotation mechanism of injuries and investigated the corresponding injury patterns. Using material testing machines on feet externally rotated to failure, the authors demonstrated that short oblique fractures of the distal fibula could be sustained in pronated feet, while exerting an additional external lateral force (abduction) to the foot resulted in a high fibular fracture [11]. Furthermore, Kwon et al. recreated the same LH methodology for SER attempting to reproduce the same results. Of all 10 cadaver specimens used, although some injury findings were consistent with the LH description of injuries, none of the cadavers demonstrated the complete sequence of injuries described by LH [12]. Hence, the reliability and reproducibility of the LH classification for predicting injuries have been recently called into question, again mandating the need for a new improved classification system.

Recently, a new innovative method for the assessment of mechanism of injury in ankle fractures was adopted. Kwan et al. investigated the mechanisms of injury of ankle fracture on YouTube videos and compared them to the corresponding LH class on radiographs obtained from individuals posting the video clips. The authors reported a 58% accuracy of LH classification in predicting the mechanism of injury. The 5 cases with SAD mechanisms of injuries completely corresponded with the LH classification. However, of the 7 PER mechanisms of injury, only 2 (29%) matched the LH classification [13]. Similarly, Rodriguez et al. reviewed YouTube videos of ankle fractures and compared their consistency with the LH versus AO classifications. Of the 14 PER mechanisms, only 5 corresponded with LH PER fracture pattern. Overall, the LH classification correctly predicted 65% of injuries, compared with 81% when the AO classification was used [14]. The AO classification has a higher reliability and reproducibility than the LH classification system and is easier to implement [15]. Nevertheless, the AO classification remains a radiologic classification compared to the LH classification, which is clinically based.

In the operative versus conservative management of ankle fractures, varying results were reported in the literature. While a longer recovery period was reported in the operative management of ankle fractures [16], no difference in the long term clinical outcome was found [17, 18]. Nevertheless, better radiological outcomes (talocrural angle compared to the normal ankle) were demonstrated in the operative groups [18]. However, Makwana et al. reported better functional outcomes in the operative group [19]. In comparing the surgical and conservative management of 185 Weber B ankle fractures, both treatment groups showed good clinical outcomes demonstrated by similar Olerud, AOFAS, and VAS scores [20].

The outcomes of operative and nonoperative management of TFL injuries have also been investigated. In our case report the patient suffered a grade 3 TFL tear. Conservative management of lateral ligaments injuries is preferred over surgical management in acute ligament injuries. It involves a 3-week cast immobilization either via a below knee cast followed by 3 months of proprioceptive rehabilitation or through functional management using supported weight bearing with external support and RICE protocol followed by rehabilitation [21, 22]. The latter protocol shows superiority in clinical outcomes [23]. However, 20% of acute ankle lateral ligament injuries fail conservative treatment, in which patients develop chronic symptoms of instability requiring surgical intervention [24, 25]. Several procedures have been described in the treatment of chronic lateral ankle instability, which can be classified into anatomic or nonanatomic (tenodesis stabilization) repairs. Choice of procedure can be made depending on quality of local tissue, extent of injury, and surgeon's preference [26]. A modified Broström technique, used in our case, is chosen if local ligaments were not attenuated and were of good quality [27, 28]. In tenodesis stabilization, the multiple tendon graft configurations can restrict motion. Hence, the hindfoot and ankle biomechanics could be affected [29].

Although the MRI has been widely used in diagnosing ankle lateral ligaments injuries in patients with chronic instability, evidence shows the anterior drawer test to be sufficient. The sensitivity and specificity of the anterior drawer test in detecting ATFL injuries are 73% and 97%, respectively. Finding a skin dimple on a positive anterior drawer test increases the predictive value to 94% [30]. Even though the LH classification has been classically used in predicting ankle injury patterns, a physical examination of ankle stability along with the advances in medical imaging does not promote relying on the LH classification, especially in light of the reported inaccuracy of the LH predictions.

In conclusion, although the LH classification classically served an important role in predicting ankle ligaments injury, many reported injuries have contradicted it. Future research should focus on producing a more reliable classification for predicting ankle ligament injuries based on the mechanism of injury. To date, the AO classification remains superior and should be more incorporated into clinical practice.
